# The Mechanisms of Fur Development and Color Formation in American Mink Revealed Using Comparative Transcriptomics

**DOI:** 10.3390/ani12223088

**Published:** 2022-11-09

**Authors:** Lidong Wang, Shengyang Zhou, Guangshuai Liu, Tianshu Lyu, Lupeng Shi, Yuehuan Dong, Shangbin He, Honghai Zhang

**Affiliations:** 1 College of Life Sciences, Qufu Normal University, Qufu 273165, China; 2 College of Wildlife and Protected Area, Northeast Forestry University, Harbin 150040, China

**Keywords:** RNA-seq, mink, tyrosinase, fur, coat color

## Abstract

**Simple Summary:**

The American mink is a fur animal that has been successfully domesticated by humans. The fur products made of mink are fine, soft and strong, plush and glossy, which are deeply loved by people. The original color of farmed mink is dark brown, but people are not satisfied with a single color of fur products. After more than one hundred years of breeding, a variety of colorful fur colors have been produced. Because white is easy to dye and black can be paired with anything, these two mink species are the most widely farmed. However, the molecular mechanisms of the color formation and fur development of these two minks remain unclear. Through skin transcriptome analysis of these two minks, we obtained the molecular mechanism of their coat color formation and fur development. The expression of tyrosinase and tyrosinase-related protein genes is the main reason for the formation of coat color. The keratin associated protein gene is the main regulatory gene for fur development. The research in this paper can provide data support for good breeding of mink.

**Abstract:**

American mink fur is an important economic product, but the molecular mechanisms underlying its color formation and fur development remain unclear. We used RNA-seq to analyze the skin transcriptomes of young and adult mink with two different hair colors. The mink comprised black adults (AB), white adults (AW), black juveniles (TB), and white juveniles (TW) (three each). Through pair comparison and cross-screening among different subgroups, we found that 13 *KRTAP* genes and five signaling pathways (the JAK–STAT signaling pathway (cfa04630), signaling pathways regulating pluripotency of stem cells (cfa04550), ECM–receptor interaction (cfa04512), focal adhesion (cfa04510), and the Ras signaling pathway (cfa04014)) were related to mink fur development. We also found that members of a tyrosinase family (*TYR*, *TYRP1*, and *TYRP2*) are involved in mink hair color formation. The expression levels of TYR were higher in young black mink than in young white mink, but this phenomenon was not observed in adult mink. Our study found significant differences in adult and juvenile mink skin transcriptomes, which may shed light on the mechanisms of mink fur development. At the same time, the skin transcriptomes of black and white mink also showed differences, with the results varying by age, suggesting that the genes regulating hair color are active in early development rather than in adulthood. The results of this study provide molecular support in breeding for mink coat color and improving fur quality.

## 1. Introduction

The *American mink* (*Neovison vison*) is one of the major farmed species of Mustelidae and one of the world’s major suppliers of quality fur [1]. Mink are semi-aquatic, so their coatis thick and waterproof, which protects them from the cold and allows them to more easily move in the water [2]. In the early 19th century, wild mink populations declined rapidly due to the economic value of their pelts; thus, during this time, the first mink farms were established by T.D. Phillips and W. Woodcock [3]. Farmed mink was originally the standard dark brown, but with genetic mutations and advances in farming techniques, more than 100 color types have become available. Short-haired black mink and red-eyed white mink are the most common breeds, as black can be worn with almost any color of clothing, and white can be dyed in any color.

The characteristics displayed by species can be divided into qualitative characteristics and quantitative characteristics, with coat color as a qualitative characteristic. A characteristic is considered qualitative when there is a noncontinuous quantitative change between different phenotypes of the same trait, which is dominated by a few decisive genes [4]. American mink has a variety of coat colors, which are each regulated by different genes. Thus far, researchers have identified coat color regulatory genes of mink of seven different coat colors (Aleutian [5], Albino [6], Palomino [7], Silverblue, Hedlund White [1], Moyle Brown [8], and Himalayan [9]), which are *LYST* (lysosomal trafficking regulator), *TYR* (tyrosinase, in Albino and Himalayan), *TYRP1* (tyrosinase-related protein 1), *MLPH* (melanophilin), *MITF* (microphthalmia-associated transcription factor), and *RAB38* (Ras-related protein-38). However, these studies have only focused on changes in gene sequences, and little attention has been paid to changes in gene expression.

In addition, the economic value of mink fur depends not only on the color but also on the overall quality (length, density) of the fur. These characteristics, associated with hair follicle development, are quantitative characteristics. The development and growth of mink hair cannot be studied independently of the hair follicle, which is found in the skin [10]. Hair follicles undergo differentiation, proliferation, and apoptosis periodically and are regulated by a variety of signaling factors [11]. Most of these key signaling molecules belong to the Wnt (wingless-related), Shh (sonic hedgehog), BMP (bone morphogenetic protein), FGF (fibroblast growth factor), TGF (transforming growth factor), and Notch signaling pathways [12]. By comparing and analyzing the skin transcriptomes of mink during development and maturity, it is possible to reveal the key genes and pathways involved in the development of mink skin.

Therefore, in this study, we sought to reveal the mechanisms underlying fur development and color determination in two kinds of mink by conducting transcriptome sequencing of skin tissues from adult and juvenile individuals for short-haired black mink and red-eyed white mink. In addition, PCA analysis, differential expression analysis, and enrichment analysis were also conducted. Comparisons were conducted between different groups, wherein the comparison between the adult and juvenile groups focused on discovering the genes and pathways linked with the development of mink fur, and the comparison between the black and white groups explored the mechanisms determining coat color. There are two main new findings from our study. First, we identified 13 keratin-associated protein genes and 5 signaling pathways that are important for fur development in American mink. Second, we found that three members of the *TYR* gene family (*TYR*, *TYRP1*, and *TYRP2*) are associated with the formation of different coat colors in mink. Based on the expression of *TYR* in the different groups, we hypothesized that the red-eyed white mink hair color may be caused by the OCA1B type of ocular skin albinism. We also found that short-haired black mink hair color is associated with higher expression of members of the *TYR* gene family. The results of this study provide a more comprehensive overview of the key genes and pathways influencing characteristics of economic value in mink fur, laying a foundation for mink breeding, including for the cultivation of new colors.

## 2. Materials and Methods

### 2.1. Sample Collection and Preparation

Twelve healthy young (10-day-old) white mink, adult white mink, young (10-day-old) black mink, and adult black mink (three of each type) were selected as samples from a mink farm in Shandong province (Figure S1). The white juvenile and adult mink were of the same breed, and the black juvenile and adult mink were of another breed. Each mink came from a different family, and all were kept under the same conditions. All sampling procedures and experimental methods were approved by the Qufu Normal University Institutional Animal Care and Use Committee (No. 2022033), Qufu, China. Two pieces of skin (1.0 cm in diameter) were collected from the back via punch skin biopsy under local anesthesia and immediately placed in liquid nitrogen. Young white skin samples were numbered TW1, TW2, and TW3. Adult white skin samples were numbered AW1, AW2, and AW3. The young black skin samples were numbered TB1, TB2, and TB3. Adult black skin samples were numbered AB1, AB2, and AB3.

### 2.2. RNA Quantification and Qualification

Total RNA was extracted from the 12 skin samples using the RNeasy Mini Kit (QIAGEN, Hilden, Germany). Total amounts and integrity of RNA were assessed using the RNA Nano 6000 Assay Kit of the Bioanalyzer 2100 system (Agilent Technologies, Santa Clara, CA, USA).

### 2.3. Library Preparation for Transcriptome Sequencing

Sequencing libraries were generated using the NEBNext Ultra RNA Library Prep Kit for Illumina (New England Biolabs, Ipswich, MA, USA) following the manufacturer’s recommendations, and index codes were added to attribute sequences to each sample. Total RNA was used as input material for mRNA purification using oligo-attached poly-T. Divalent cations were fractured at elevated temperatures in a first-chain synthetic reaction buffer (5X). First-strand cDNA was synthesized using random hexamer primers and M-MuLV reverse transcriptase, and the RNA was degraded using RNaseH. The second strand of cDNA was then synthesized using DNA polymerase I and dNTP. The resulting overhangs were converted to blunt ends by exonuclease/polymerase activity. After adenylation of the 3′ end of the DNA fragment, an adaptor with a hairpin ring structure was attached in preparation for hybridization. The AMPure XP system (Beckman Coulter, Brea, CA, USA) from Beckman Coulter, Brea, CA, USA, was used to purify the library fragments with a selection of 370–420 bp cDNA fragments. PCR amplification was performed, the PCR product was purified with AMPure XP microbeads, and the library was finally obtained.

The constructed libraries were tested to ensure their quality. Following construction, the DNA contents of the libraries were quantified using a Qubit2.0 fluorescence analyzer and then diluted to 1.5 ng/uL, and the quality was assessed using an Agilent 2100 bioanalyzer. Following the size selection of inserts, qPCR was used to accurately quantify the effective concentration of the library (the effective concentration of the library was higher than 2 nM) to ensure high quality.

### 2.4. Clustering and Sequencing

Following validation, the different libraries were pooled according to the effective concentration and the target amount of data of the machine and then sequenced using the Illumina NovaSeq 6000 for the generation of 150 bp paired-end reads. The basic principle of sorting is that composition and sorting occur simultaneously (composition sorting). Each of the four fluorescently labeled dNTPs, DNA polymerase, and splicing primers were added to the sequenced flow cells for amplification. When the sequence cluster extends the complementary chain, each fluorescently labeled dNTP releases corresponding fluorescence, which is captured by the sequencer, and the optical signal is converted into a sequencing peak through computer software to obtain the sequence information of the fragment to be measured.

### 2.5. Quality Control

Image data, measured using a high-throughput sequencer, were converted to sequence data (read) via CASAVA base recognition. Raw data in FastQ format (raw reads) were first processed using an internal Perl script. In this step, clean reads were obtained by removing reads containing adapters, reads containing N bases, and low-quality reads from the raw data. The Q20, Q30, and GC contents of clean data were also calculated. All downstream analyses were based on high-quality clean data.

### 2.6. Read Mapping to the Reference Genome

The reference genome was accessed from the National Center for Biotechnology Information (NCBI; https://www.ncbi.nlm.nih.gov accessed on 6 March 2022, GCF_020171115.1). The reference genome index was constructed using Hisat2 (V2.0.5), and the paired clean reads were compared with the reference genome using Hisat2 (V2.0.5) [13]. We chose Hisat2 as the mapping tool because it can generate a splicing connection database from a gene model annotation file, and its mapping effects are superior to those of other nonsplicing mapping tools.

### 2.7. Novel Transcripts Prediction

The mapped reads of each sample were assembled using StringTie [14] (v1.3.3b) in a reference-based approach. StringTie uses a novel network flow algorithm with optional ab initio assembly steps to assemble and quantify full-length transcripts representing multiple spliced variants of each gene loci.

### 2.8. Quantification of Gene Expression Level

The featureCounts v1.5.0-p3 tool was used to count the read numbers mapped to each gene [15]. The FPKM value of each gene was then determined based on the length of the gene and the mapped read count [16]. FPKM, the expected number of fragments per thousand base pairs of transcriptional sequence, is the parameter most frequently used for estimating gene expression level, and it takes into consideration the effects of sequencing depth and gene length corresponding to the read count.

### 2.9. Differential Expression Analysis

Differential expression analysis of two groups was performed using the DESeq2 R package (1.20.0) [17]. DESeq2 provides arithmetical procedures for determining differential expression in digital gene expression data using a model based on the negative binomial distribution. To control the error detection rate, we used Benjamini and Hochberg’s method for the adjustment of the obtained *p*-values. Padj ≤ 0.05 and |log2(foldchange)| ≥ 1 were set as the thresholds for significantly differential expression.

### 2.10. GO and KEGG Enrichment Analysis of Differentially Expressed Genes

The gene ontology (GO) enrichment analysis of differentially expressed genes was performed using the clustering map R package (3.8.1), which corrects for gene length bias. We considered a *p*-value less than 0.05 to indicate significant enrichment in a particular GO category for the differentially expressed genes. KEGG is a database resource for understanding the advanced functions and uses of biological systems, such as cells, organisms, and ecosystems, from large-scale molecular datasets generated from molecular-level information, particularly information from genome sequencing and other high-throughput experimental techniques (available online: http://www.genome.jp/kegg/ accessed on 20 March 2022) [18]. We used the Cluster Profile R Package (3.8.1) [19] to detect the statistically significant enrichment of differentially expressed genes in the KEGG pathways. The reference species selected for GO and KEGG bioinformation analysis were domestic dogs (*Canis lupus familiaris*).

### 2.11. Verification of Differentially Expressed Genes by qPCR

To verify the accuracy of the transcriptomic data, we used the same RNA samples as the transcriptome analysis; in total, 12 genes (*TYR*, *TYRP1*, *TYRP2*, *KRTAP1-3*, *KRTAP2-3*, *KRTAP3-3*, *KRTAP4-2*, *KRTAP4-12*, *KRTAP9-2*, *KRTAP10-7*, *KRTAP12-1,* and *KRTAP29-1*) identified via the above methods were characterized by qPCR analysis. We used Primer Express3.0 to design primers for qPCR that spanned an exon–exon boundary, and the β-actin gene was selected for use as an internal control. The ABI7500 Real-time system (Applied Biosystems, Waltham, MA, USA) and the SYBR select Master Mix Kit (ABI Life, Waltham, MA, USA) were used for qPCR with first-strand cDNA as a template. The PCR amplification procedure consisted of incubation at 95 °C for 10 s, followed by 40 cycles of 95 °C for 15 s and 34 °C for 62 s. The product fluorescence was detected after each cycle. The qPCR analysis of each gene included three biological replicates, and primer information is shown in Table S1. Relative mRNA expression was analyzed using the 2^−ΔΔCT^ method [20].

## 3. Results

### 3.1. Data Quality Control and Novel Gene Prediction

After filtering out low-quality reads and removing the adaptor sequences, we finally obtained 80.71 Gb of clean data (Table 1). The effective average GC ratio was 51.9%. The proportion of bases with quality scores above Q30, which corresponds to a base recognition accuracy rate of 99.9%, was above 93.57%, and the ratio of each sample that aligned to the reference genome ranged from 94.69 to 96.58% (Table S2). Additionally, most reads were mapped to the exon regions (Table S3), which overall indicates the good quality of the sequencing data and its potentially high utilization. We predicted and assembled 1943 new genes using the software and functionally annotated them using Pfam (Table S4). These data were used for the analysis of gene expression.

### 3.2. Quantitative Analysis of Gene Expression

**Figure 1:**
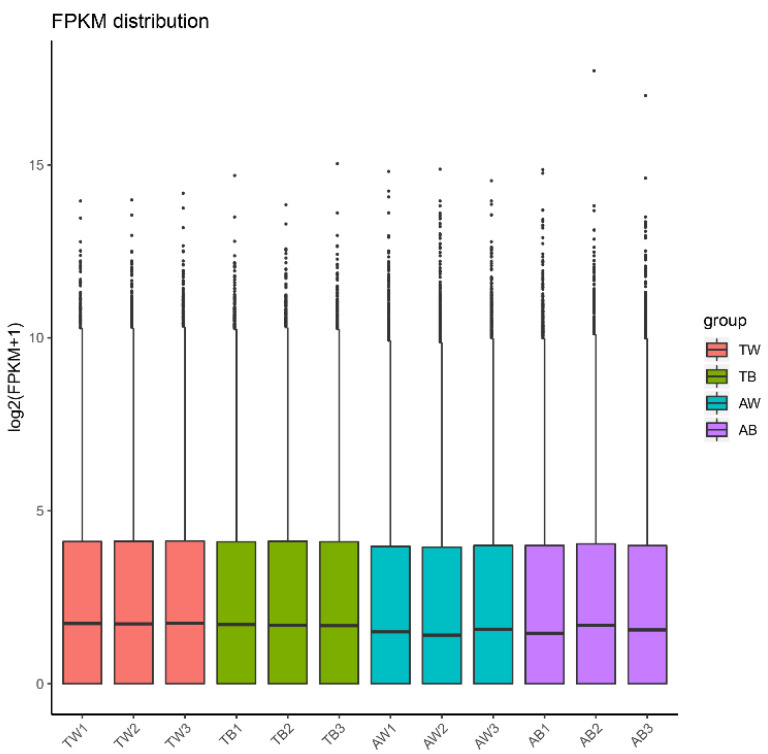
Comparison of the distribution of gene expression levels in different samples. The abscissa is the sample name, and the ordinate is log2 (FPKM + 1).

**Figure 2:**
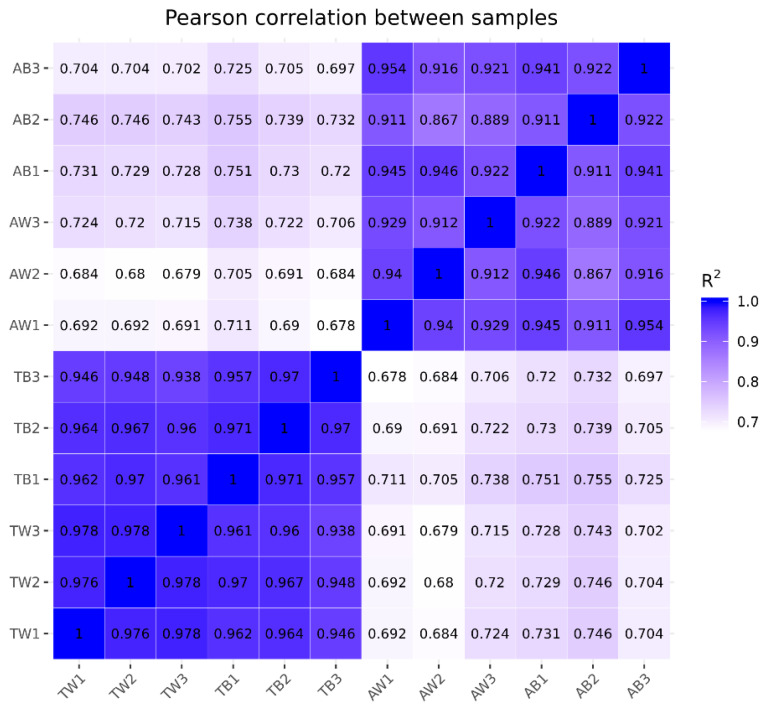
Sample correlation heat map. The horizontal and vertical coordinates are the squared correlation coefficients of each sample.

**Figure 3:**
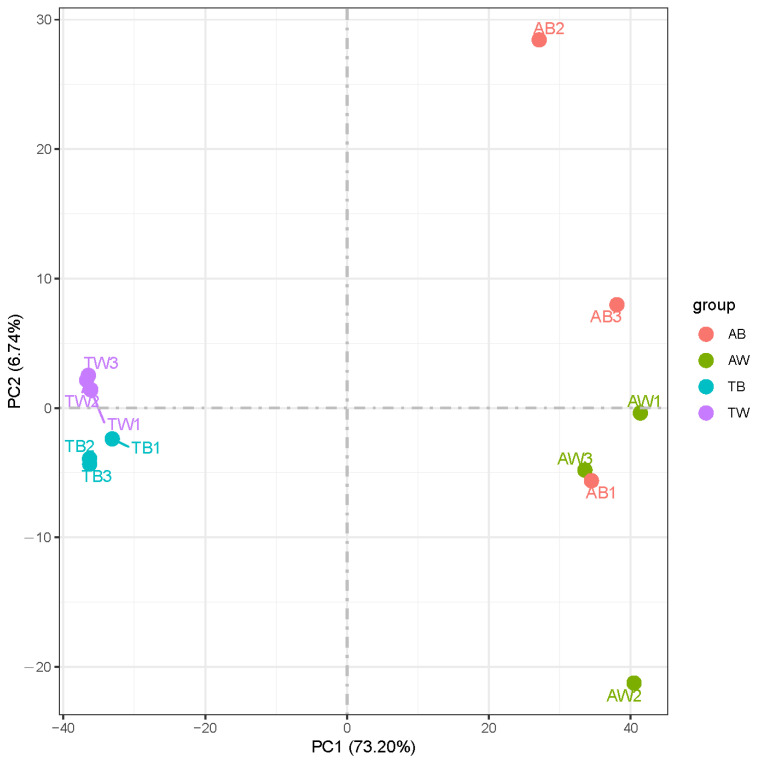
Principal component analysis (PCA). The abscissa is the first principal component, and the ordinate is the second principal component.

The gene expression levels in each sample were quantitatively analyzed and then combined to obtain an expression matrix of all samples (Table S5). After the expression values of all genes in each sample (FPKM) were calculated, the distribution of gene expression levels in different samples was displayed in a box graph (Figure 1). The correlation of gene expression levels between samples is an important index to test whether the experiment is reliable and the sample selection is reasonable. The heat map of the correlation between samples shows that they can be divided into two groups according to age factors, and the Pearson correlation coefficient R^2^ within the groups is greater than 0.8 (Figure 2). PCA analysis of gene expression values (FPKM) in all samples was conducted. The results show that the samples were divided into adult and juvenile groups at the PC1 level and further grouped according to coat color at the PC2 level (Figure 3). This shows the high reliability of our sample collection and grouping.

### 3.3. Differentially Expressed Genes (DEGs) in American Mink Skin

**Figure 4:**
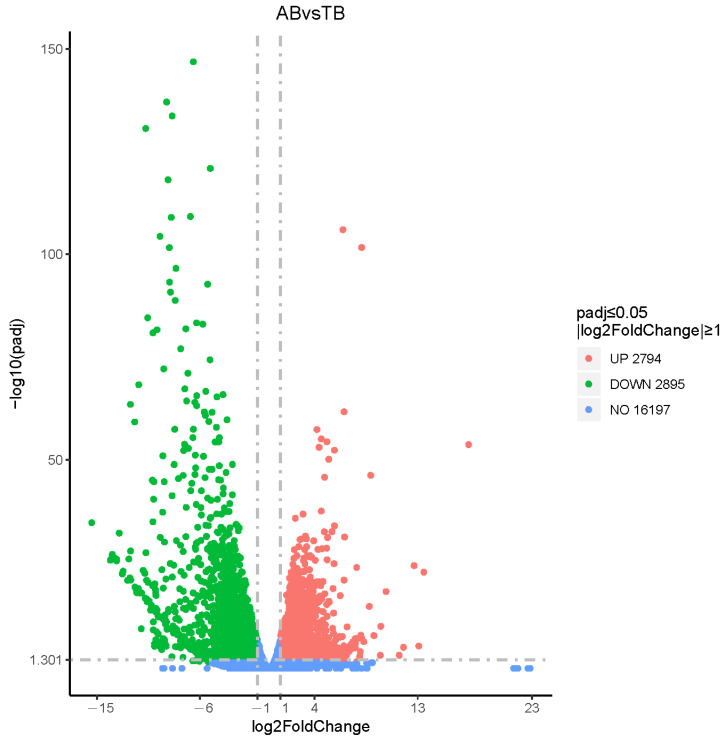
Volcano map of differentially expressed genes in comparison of different combinations. The abscissa is the log2FoldChange value, the ordinate is the −log10 *p*-value, and the blue dashed line represents the threshold line of differential gene screening criteria.

**Figure 5:**
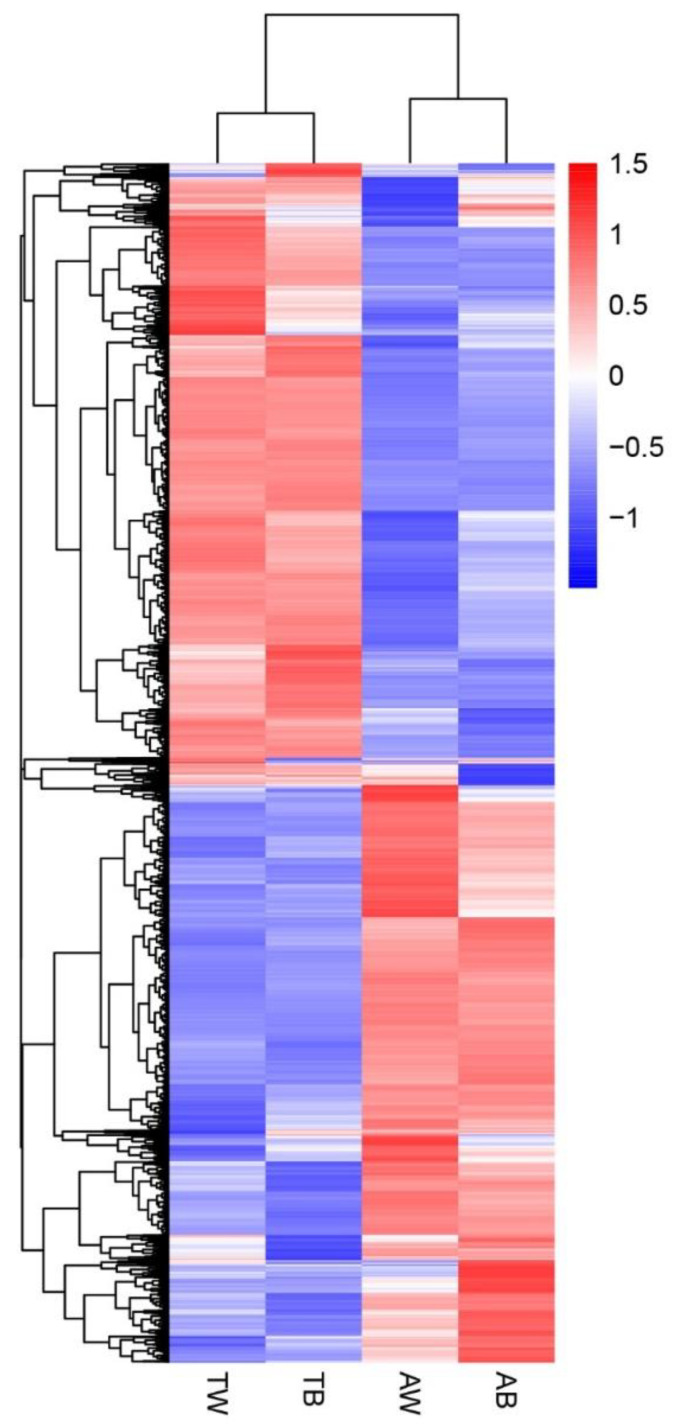
Cluster heat map of differentially expressed genes. The abscissa is the sample name, and the ordinate is the normalized value of the differential gene FPKM. The darker the red, the higher the expression level, and the darker the blue, the lower the expression level.

**Figure 6:**
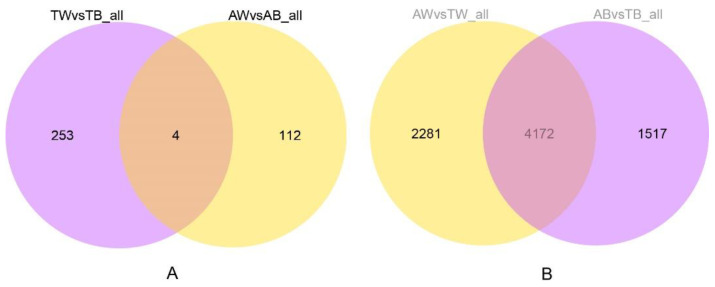
Venn diagram of differentially expressed genes in mink of (**A**) different hair colors showing the overlap between two age groups and (**B**) different age groups showing the overlap between two coat color groups.

To reveal the genes of American mink differentially expressed in the fur development group (AB vs. TB, AW vs. TW) and coat color group (AB vs. AW, TB vs. TW), four combinations were compared and analyzed (Figure 4 and Figure S2, Figure S3, Figure S4). In terms of fur development, there were 6453 and 5689 differentially expressed genes found in the white and black groups, respectively. In the coat color difference combination, there were 116 differentially expressed genes in the adult group, and 257 differentially expressed genes in the juvenile group. The differentially expressed genes in all comparison groups were combined as differential gene sets, on which cluster analysis was carried out, and genes with similar expression patterns were clustered together (Figure 5). In addition, we plotted differential gene Venn plots from pairwise combinations of the coat color group and the fur development group (Figure 6). This Venn diagram A implies that differentially expressed genes in minks of the same age and different coat colors may be related to coat color. We took the intersection of differentially expressed genes between the young group and the adult group to further pinpoint the candidate genes for coat color. Similarly, the intersection derived from the Venn diagram B is the fur development gene of the mink. There were only four differentially expressed genes common to adults and juveniles of different coat colors. Unfortunately, this included two noncoding RNAs for *SCD5* (stearoyl-CoA desaturase 5) and *RSPH4A* (radial spoke head component 4A), which would not be associated with the coat color difference. There were 4172 differentially expressed genes common to both the white and black groups of different ages. These differentially expressed genes may be related to fur development in mink.

### 3.4. Analysis of GO and KEGG Pathways

To further investigate the biological relevance of all DEGs, we performed GO analysis of the DEGs. To ensure the enrichment results were more likely to reflect real phenomena, we examined significantly altered genes within the intersection of the fur development group in the two comparisons (AW vs. TW and AB vs. TB) and obtained 73 terms that were significantly enriched, including 18 biological process (BP) categories, 17 cellular component (CC) categories, and 38 molecular function (MF) categories (Table S6). Interestingly, the terms of keratin filaments (GO:0045095) related to fur development were also included. A total of 13 genes, including *KRTAP1-3*, *KRTAP2-3*, *KRTAP3-3*, *KRTAP4-2*, *KRTAP4-5*, *KRTAP4-11*, *KRTAP4-12*, *KRTAP9-2*, *KRTAP10-7*, *KRTAP10-8*, *KRTAP10-12*, *KRTAP12-1*, and *KRTAP29-1*, were finally identified, all of which were downregulated. Similarly, the enrichment results corresponding to the intersection in the comparison of coat color groups (AW vs. AB and TW vs. TB) were considered, for which there were only three GO terms: structural molecule activity (GO:0005198), sulfotransferase activity (GO:0008146), and transferase activity (GO:0016782).

**Figure 7:**
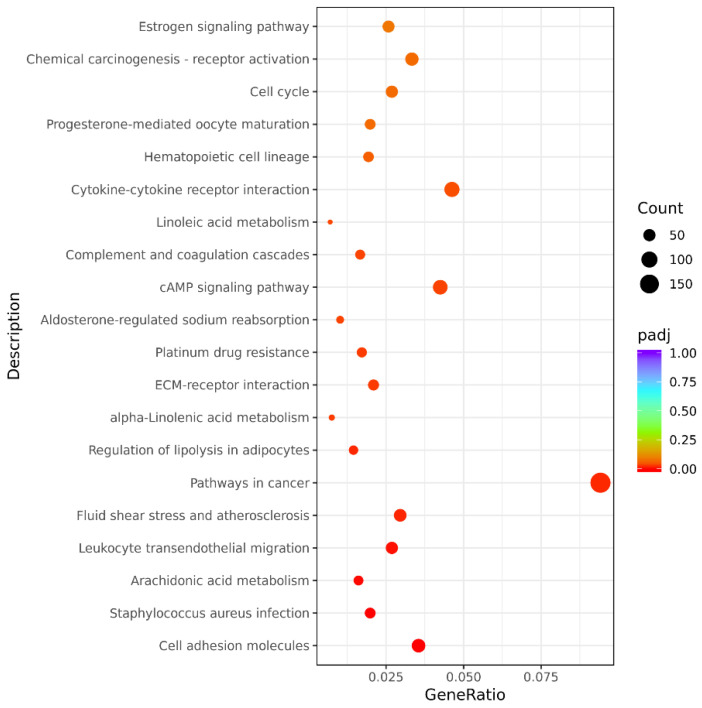
Scatter plot of KEGG enrichment analysis for differentially expressed genes (AB vs. TB). The abscissa is the ratio of the number of differentially expressed genes annotated to the KEGG pathway to the total number of differentially expressed genes, and the ordinate is the KEGG pathway.

The results of the KEGG annotation analysis of the differentially expressed genes between groups are shown in Figure 7 and Figure S5, Figure S6, Figure S7. Similar to the above analysis, we obtained results related to the metabolic pathways for both fur development and coat color differences. There were 35 cross-enriched related metabolic pathways in different age combinations of the same coat color, among which the JAK–STAT signaling pathway (cfa04630), signaling pathways regulating pluripotency of stem cells (cfa04550), ECM–receptor interaction (cfa04512), focal adhesion (cfa04510), and Ras signaling pathway (cfa04014) were related to fur development (Table S7). Interestingly, only one metabolic pathway was identified via the intersection of enrichment results of different coat colors at the same age: tyrosine metabolism (cfa00350). This pathway comprises five differentially expressed genes, namely *DCT*, *TYR*, *AOX4*, *GSTZ1*, and *ADH5*.

### 3.5. qPCR Validation of Differential Gene Expression among Groups of American Mink

The differential expression of genes, as identified from the transcriptome data, was validated by qPCR analysis. There were two groups of genes that needed to be verified by qPCR experiment: nine keratin-associated protein genes in the fur development group and three genes in the hair-color-related *TYR* gene family (*TYR*, *TYRP1*, and *TYRP2*). The RNA-seq results indicated that the gene expression level of *KRTAP* was higher in young mink than in adult mink. At the same time, the *TYR* gene was highly expressed in young black mink, and *TYRP1* and *TYRP2* were highly expressed in young white mink. Key gene expression verification results (Figure S8) showed that they were consistent with RNA-seq expression patterns, which implies that the transcriptome results are reliable.

## 4. Discussion

Mink is one of the most important fur animals in the world. Previous studies have focused only on the diversity of fur color and its mechanisms of formation; however, in this study, we not only explored the mechanisms of fur color formation but also focused on key genes and pathways in the process of fur development. Three juveniles and three adults of the short-haired black mink breed and three juveniles and three adults of the red-eyed white mink were selected as our experimental samples. A comparison of skin transcriptomes between different groups was performed to achieve our research objectives. Animal coat color is a qualitative characteristic that is determined by the content of eumelanin and pheomelanin in the hair [21]. According to the International Federation of Pigment Cell Societies (IFPCS), 661 genes related to coat color have been reported to date [22]. Current research on fur development mainly focuses on the molecular mechanisms of hair follicle development, including keratin [23,24], keratin-associated proteins [25], signal pathways [26], gene families [27], and growth factors [28,29].

Transcriptome sequencing of 12 mink skin samples yielded a total of 538 million clean reads. Correlation analysis between the samples showed that the samples could be divided into two components by age, indicating that the differences in gene expression related to different coat colors are very small. Further principal component analysis (PCA) on the expression levels found differences in samples from mink of different colors. This shows that our experimental design is reasonable with respect to the aims. Differential gene expression analysis showed that the differentially expressed genes shared by adult black and white mink (AB vs. AW) and juvenile black and white mink (TB vs. TW) were not related to coat color formation. At the same time, adult black and juvenile black mink (AB vs. TB) shared too many differentially expressed genes (4172) with adult white and juvenile white mink (AW vs. TW) for the genes related to fur development to be identified.

Therefore, we searched for genes related to hair color formation and fur development through GO enrichment and KEGG enrichment analysis. The analysis showed that differentially expressed genes were enriched in 73 GO terms, and the most significant term we identified as being related to fur development was keratin filaments (GO:0045095). Keratin filaments are intervening filaments made of keratin that are found in various epithelial cells [30]. This GO term is associated with 13 keratin-associated protein genes (*KRTAP*), all of which were downregulated in adult mink. The product of *KRT* is the main protein that makes up the outer layers of hair and skin [31,32]. It has been found that mutations in the *KRT* gene can lead to changes in hair shape in dogs [33], cats [34], and mice [35], resulting in curly hair. Mutations in the *KRT* gene could also cause hereditary skin diseases in humans, such as ichthyosis, congenital tachycardia, and palmoplantar epidermolytic keratosis [36,37]. *KRTAP* can be divided according to the composition of amino acids in the encoded protein as homocysteine (HS-KAPS) and high glycine tyrosine (HGT-KAPS) [38,39]. The 13 *KRTAP* genes we identified encoded HS-KAPS. It has been reported that the *KRTAP* gene is linked with wool weight, strength, diameter, elongation, and other traits [40,41,42,43]. In addition, different numbers of cysteine-containing repeats encoded by human *KRTAP1* and *KRTAP2* genes lead to length polymorphism, which changes the interaction between *KRT* and *KRTAP* and results in differences in hair traits among individuals [44]. From these, we can infer that these 13 *KRTAP* genes are likely to play an important role in the development of mink fur.

KEGG enrichment analysis also led to the identification of five development-related signaling pathways. In mammals, the JAK–STAT pathway is the main signal transduction mechanism for a variety of cytokines and growth factors [45]. Pluripotent stem cells (PSCs) are basic cells with an indefinite self-renewal capacity, which are associated with the TGF [46] and BMP [47] signaling pathways. The extracellular matrix (ECM) is a mixture of macromolecules with complex structures and functions, which plays an important role in the morphogenesis of tissues and organs and the maintenance of cell and tissue structure and function [48]. Adhesion molecules, while catalytically inactive, can participate in this process by binding to growth factor receptors, which can initiate, integrate, or feedback adhesion-based signals [49]. Ras proteins are GTPases, which act as molecular switches in signaling pathways that regulate cell proliferation, survival, growth, migration, differentiation, and cytoskeletal activity [50]. In conclusion, we speculate that these signaling pathways may be related to mink fur development.

In addition, the tyrosine metabolism pathway in KEGG enrichment was identified as being related to hair color. Two genes in this pathway (*TYR* and *DCT*) are associated with melanogenesis. The tyrosinase gene family that regulates melanin production mainly consists of *TYR* (tyrosinase), *TYRP1* (tyrosinase-related protein 1), and *TYRP2* (dopachrome tautomerase, *DCT*) [51]. Tyrosinase is a rate-limiting enzyme in the process of melanin synthesis. The expression level and activity of *TYR* directly affect the expression of eumelanin and melanin and thus affect animal hair color [52]. *TYRP1* and *TYRP2* play a catalytic role in the ultimate steps controlling the type of melanin melanocytes produce [53]. In our results, the expression level of *TYR* was higher in young black mink than in young white mink, but this phenomenon was not observed in adult mink. Interestingly, *TYRP1* and *TYRP2* were more highly expressed in young white mink than in black mink. In adult mink, only *TYRP2* was highly expressed in black mink, while there was no significant difference in the expression of *TYR* and *TYRP1*. Therefore, it can be inferred that the regulatory genes of mink hair color play a role in early development, but there is no significant difference in gene expression levels in adults.

To further explore the influence of the tyrosinase gene family on mink hair color, we consider the formation of black and white mink hair color on a deeper level by referring to previous studies. Oculocutaneous albinism (OCA) is a disorder of melanin synthesis or transport caused by homozygous mutations in an autosomal recessive gene that is characterized by reduced pigmentation of the skin, hair, and retina [54]. There are many types of OCA, among which the *TYR* gene is the pathogenic cause of OCA1 [55]. According to our results, the *TYR* gene was underexpressed in young white mink. In most mammals, such as rabbits [56], cattle [57], rats [58], cats [59], and ferrets [60], albino phenotypes are caused by mutations in the *TYR* gene. By observing the appearance characteristics of the red-eyed white mink and the low expression of the *TYR* gene, we speculate that the defining characteristics of red-eyed white mink are linked with OCA1. Meanwhile, different mutations of the *TYR* gene lead to different degrees of abnormality in the function of the enzymatic protein expressed by the *TYR* gene. OCA1 can be divided into OCA1A and OCA1B subtypes depending on whether tyrosinase activity is completely lost [61]. We observed that although the expression level of the *TYR* gene was higher in young black mink than in white mink, there was still some expression in white mink. Therefore, the red-eye white phenotype in this study was due to OCA1B caused by a defect in *TYR*.

We observed that *TYR* was highly expressed in young black mink, which resulted in increased production of eumelanin and darker hair color. The gene *α-MSH* (alpha-melanocyte-stimulating hormone) can activate *MITF* (melanocyte-inducing transcription factor) and cause *MITF* to act on the *TYR* gene promoter to catalyze *TYR* metabolism, thus promoting the synthesis of eumelanin [62]. The *ASIP* (agouti signaling protein) can inhibit the expression of *MITF* and reduce the binding between the promoter region of the *TYR* gene and *MITF*. In this way, the activity of *TYR* will decrease, and pheomelanin will be produced [63]. Moreover, *α-MSH* can bind to *MC1R* (melanocortin 1 receptor) to activate the cAMP enzyme system and enhance the activity of *TYR* to produce eumelanin [64]. When *α-MSH* and *ASIP* competitively bind to *MC1R*, the combination of *ASIP* and *MC1R* cannot activate the whole system of the cAMP enzyme, so cAMP cannot be produced, which leads to low *TYR* activity and the production of pheomelanin [65]. We examined *ASIP*, *MC1R*, *MITF*, and *α-MSH* genes and found no significant differences in gene expression between the juvenile and adult groups with different hair colors. As a member of the *TYR* family, *TYRP2* has DOPA pigment isomerase (DT) activity and can catalyze the transformation of DOPA pigment into a 5,6-dihydroxyindole carboxylic acid (DCH-CA), thus accelerating the formation of melanin [66]. We observed that *TYR* and *TYRP2* were highly expressed in young black mink and may be responsible for the formation of black mink coat color.

## 5. Conclusions

In summary, we identified differences in skin transcriptomes between juvenile and adult mink and between black and white mink. In this study, skin transcriptomes of four groups of mink (AB, AW, TB, TW) were sequenced, and we found 13 *KRTAP* genes and five signaling pathways that are potentially involved in the fur development of mink. Meanwhile, *TYR* and *TYRP2* are closely linked to the formation of mink fur color. The results of this study can provide a reference for studying the molecular mechanisms of mink fur development and color formation.

## Figures and Tables

**Table 1:** Sequencing data statistics.

Sample	Raw-Reads	Clean-Reads	Clean-Bases	Q20	Q30	GC-pct
AB1	45,947,094	45,190,978	6.78 G	97.7	93.57	52.55
AB2	43,685,582	42,895,824	6.43 G	97.83	93.81	52.43
AB3	46,277,030	45,357,284	6.8 G	97.82	93.83	52.28
AW1	44,938,430	44,155,634	6.62 G	97.73	93.69	52.94
AW2	48,298,078	47,408,254	7.11 G	97.88	93.94	51.17
AW3	46,758,560	46,088,186	6.91 G	97.84	93.87	51.52
TB1	43,634,474	42,882,980	6.43 G	97.76	93.71	52.22
TB2	47,393,846	46,084,054	6.91 G	98.24	94.89	51.14
TB3	46,278,052	44,685,668	6.7 G	98.18	94.64	49.18
TW1	44,342,806	43,591,538	6.54 G	97.68	93.56	52.31
TW2	44,828,808	43,975,908	6.6 G	97.71	93.62	52.77
TW3	46,740,826	45,858,686	6.88 G	97.75	93.72	52.91

Q20, Q30: Represent base recognition error rates of 1% and 0.1% in the sequencing process, respectively.

## Data Availability

The datasets generated during the current study are available in the NCBI Sequence Read Archive (SRA) database. AB1-3: SRR18697439, SRR18697438, SRR18697435. TW1-3: SRR18697437, SRR18697436, SRR18697428. AW1-3: SRR18697434, SRR18697433, SRR18697432. TB1-3: SRR18697431, SRR18697430, SRR18697429.

## Supplementary Materials

The following supporting information can be downloaded at: https://www.mdpi.com/article/10.3390/ani12223088/s1, Figure S1: Juvenile black and white mink images; Figure S2: Volcano map of differentially expressed genes in comparison of different combinations (AW vs. AB); Figure S3: Volcano map of differentially expressed genes in comparison of different combinations (AW vs. TW); Figure S4: Volcano map of differentially expressed genes in comparison of different combinations (TW vs. TB); Figure S5: Scatter plot of KEGG enrichment analysis for differentially expressed genes (AW vs. AB); Figure S6: Scatter plot of KEGG enrichment analysis for differentially expressed genes (AW vs. TW); Figure S7: Scatter plot of KEGG enrichment analysis for differentially expressed genes (TW vs. TB); Figure S8: The qPCR was used to detect the expression levels of differentially expressed genes. Table S1: The qPCR primer information; Table S2: Statistical results of sample and reference genome alignment; Table S3: Reads were mapped to genomic regions for statistics; Table S4: Prediction and assemble of annotated new genes; Table S5: FPKM values of genes in all samples; Table S6: Intersection of GO Term differentially expressed genes in different age groups of two fur colors; Table S7: Intersection of signal pathway differentially expressed genes in different age groups of two fur colors.
