# Supplementary figures and images for: The Mechanisms of Fur Development and Color Formation in American Mink Revealed Using Comparative Transcriptomics
Source: Animals (Basel). 2022 Nov 9;12(22):3088. doi: 10.3390/ani12223088 (PMC9686883; doi:10.3390/ani12223088)

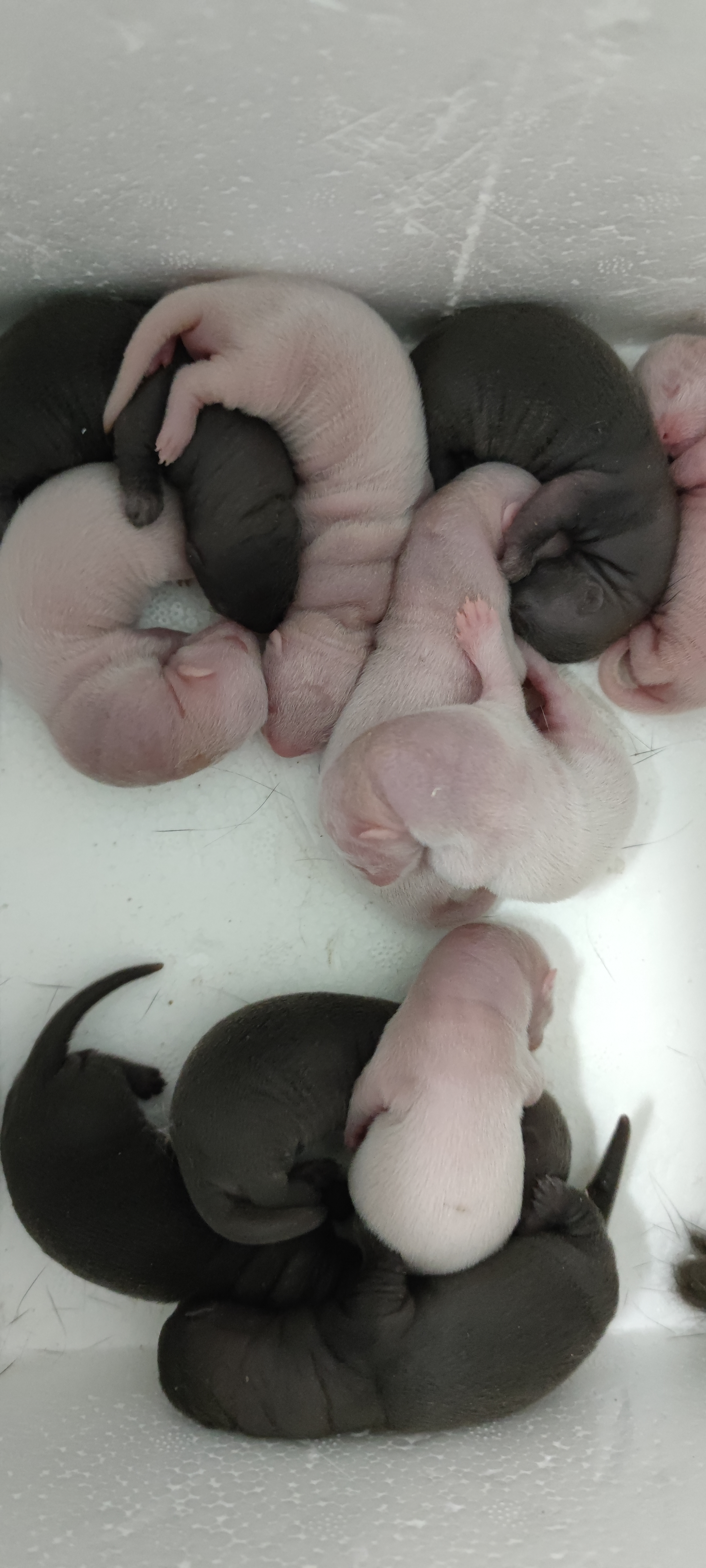

Supplement: Supplementary file 1 [file animals-12-03088-s001.zip › Supplementary/FigS1.jpg]

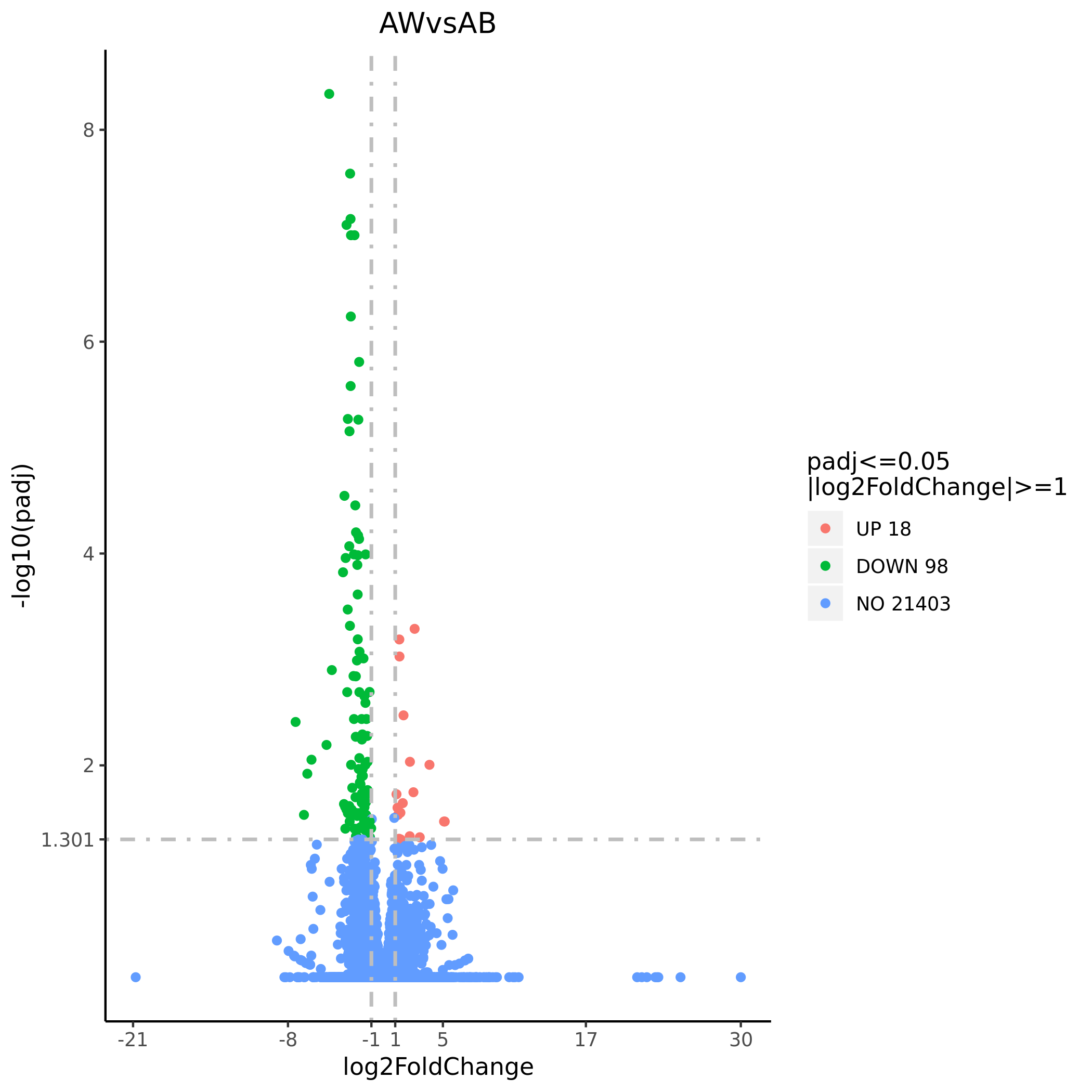

Supplement: Supplementary file 1 [file animals-12-03088-s001.zip › Supplementary/FigS2.png]

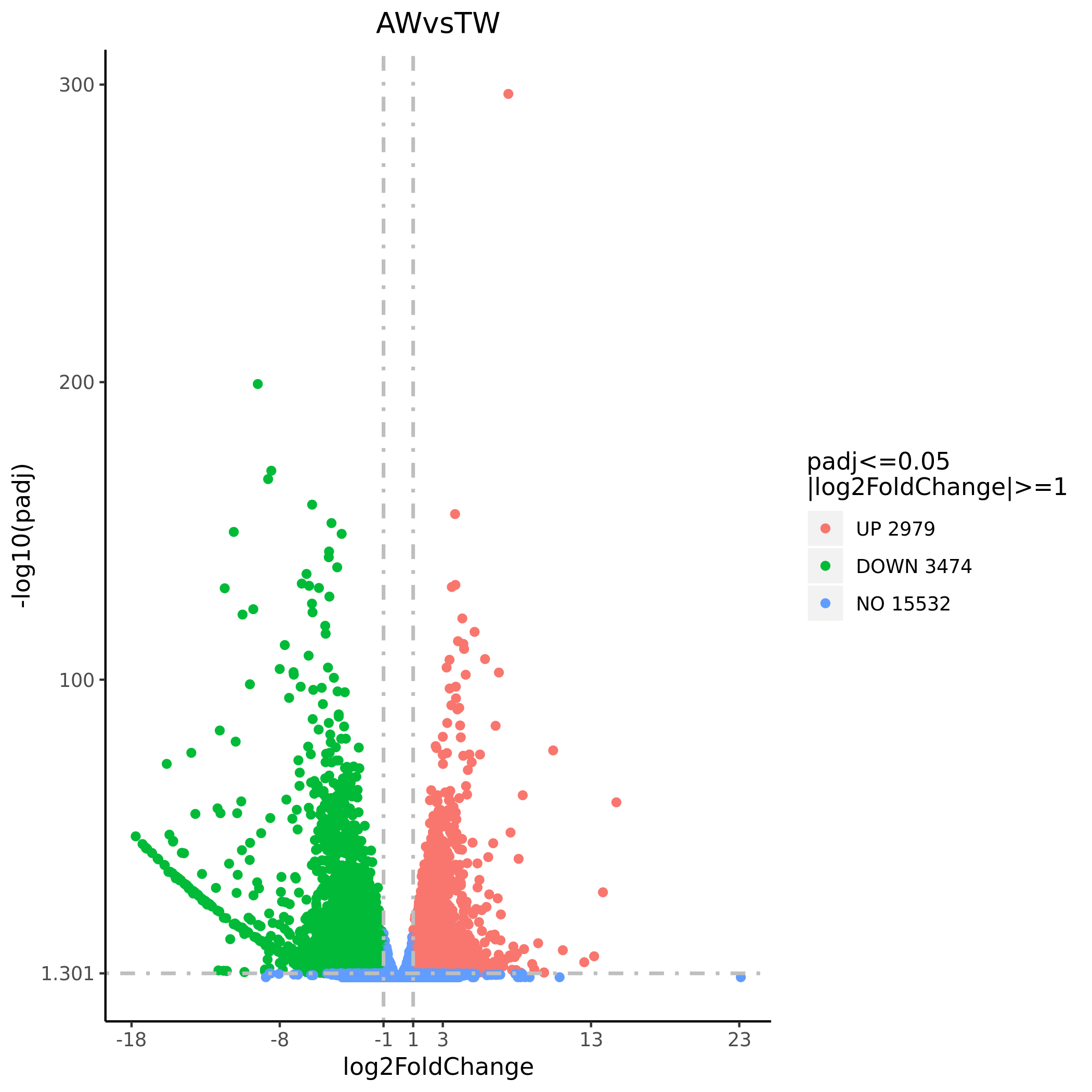

Supplement: Supplementary file 1 [file animals-12-03088-s001.zip › Supplementary/FigS3.png]

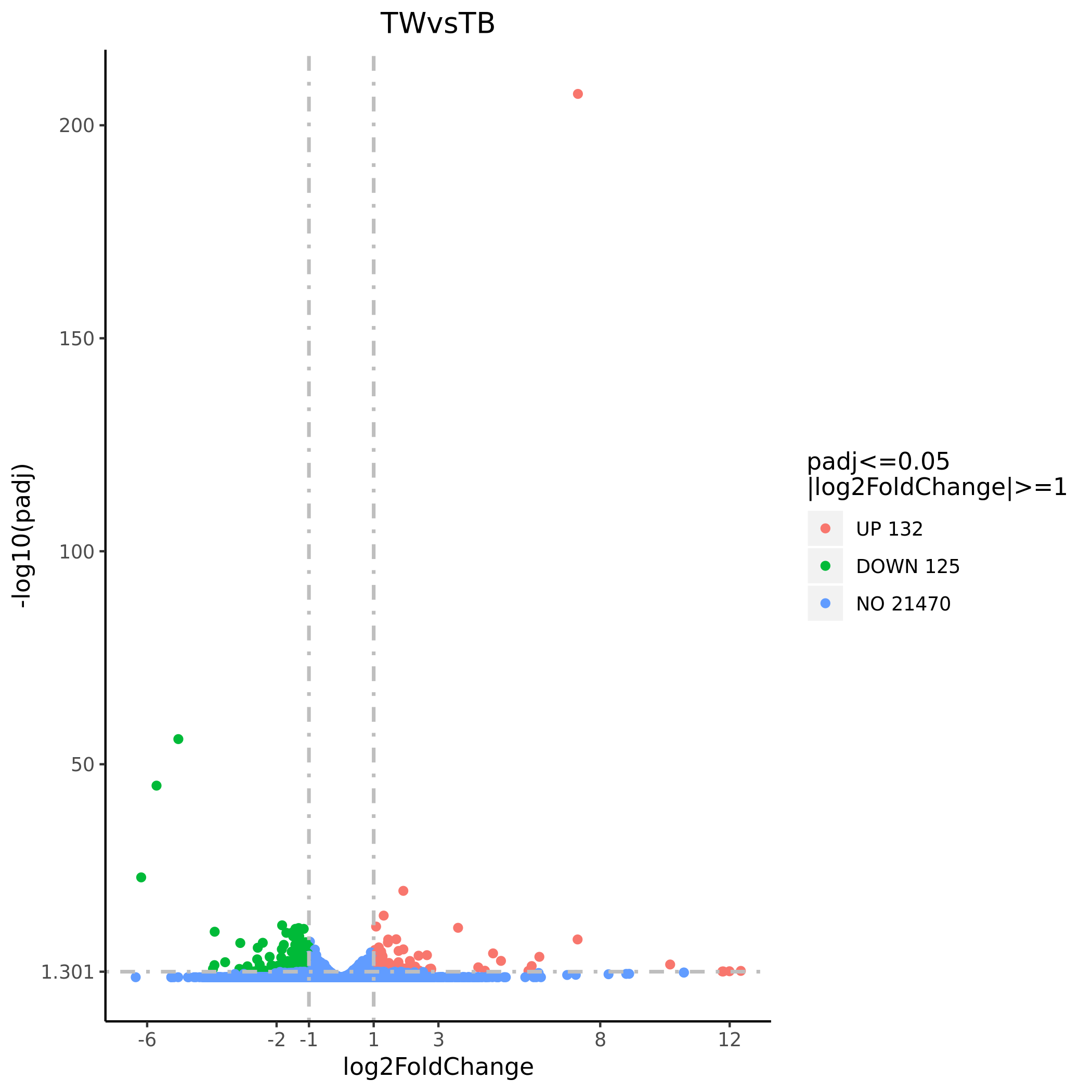

Supplement: Supplementary file 1 [file animals-12-03088-s001.zip › Supplementary/FigS4.png]

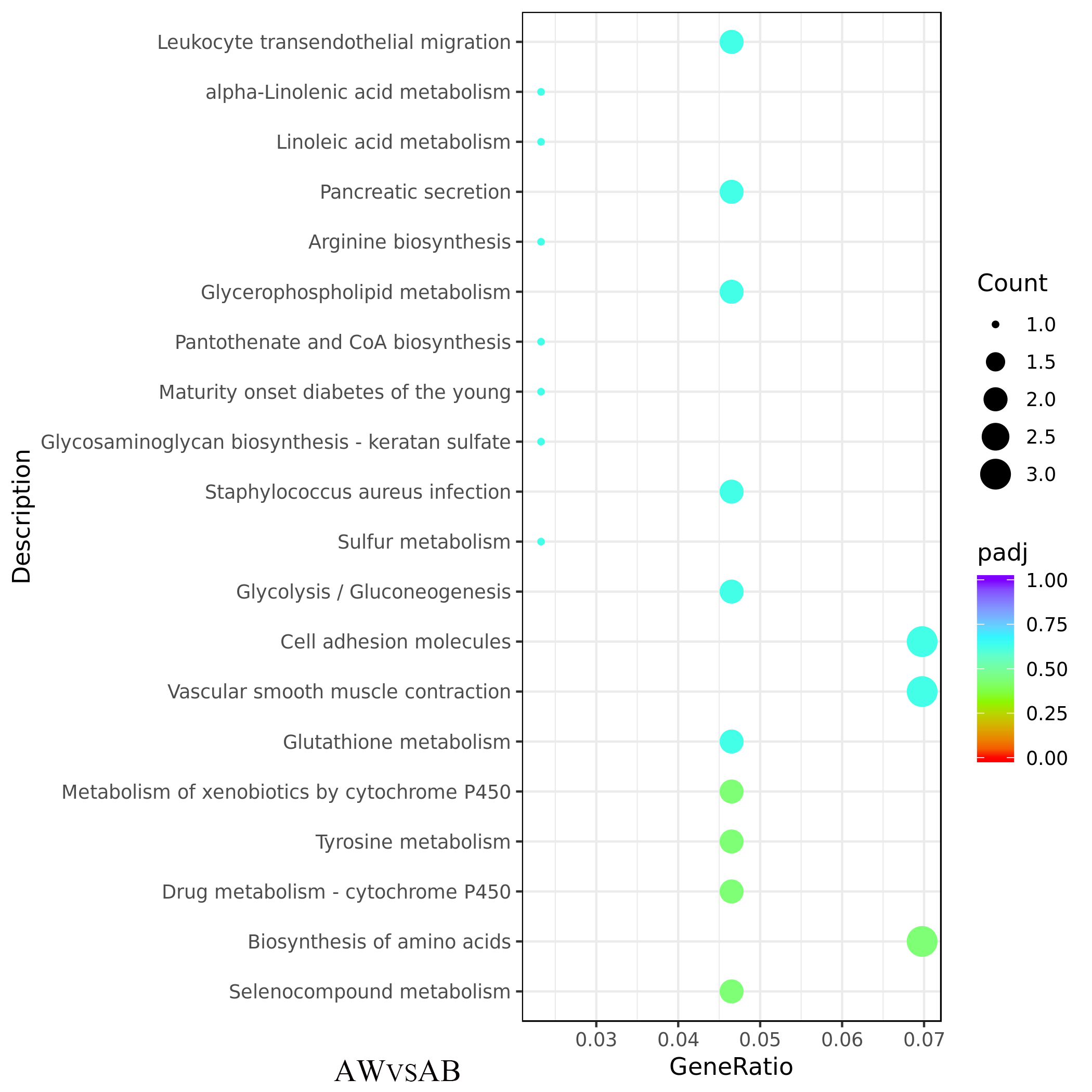

Supplement: Supplementary file 1 [file animals-12-03088-s001.zip › Supplementary/FigS5.png]

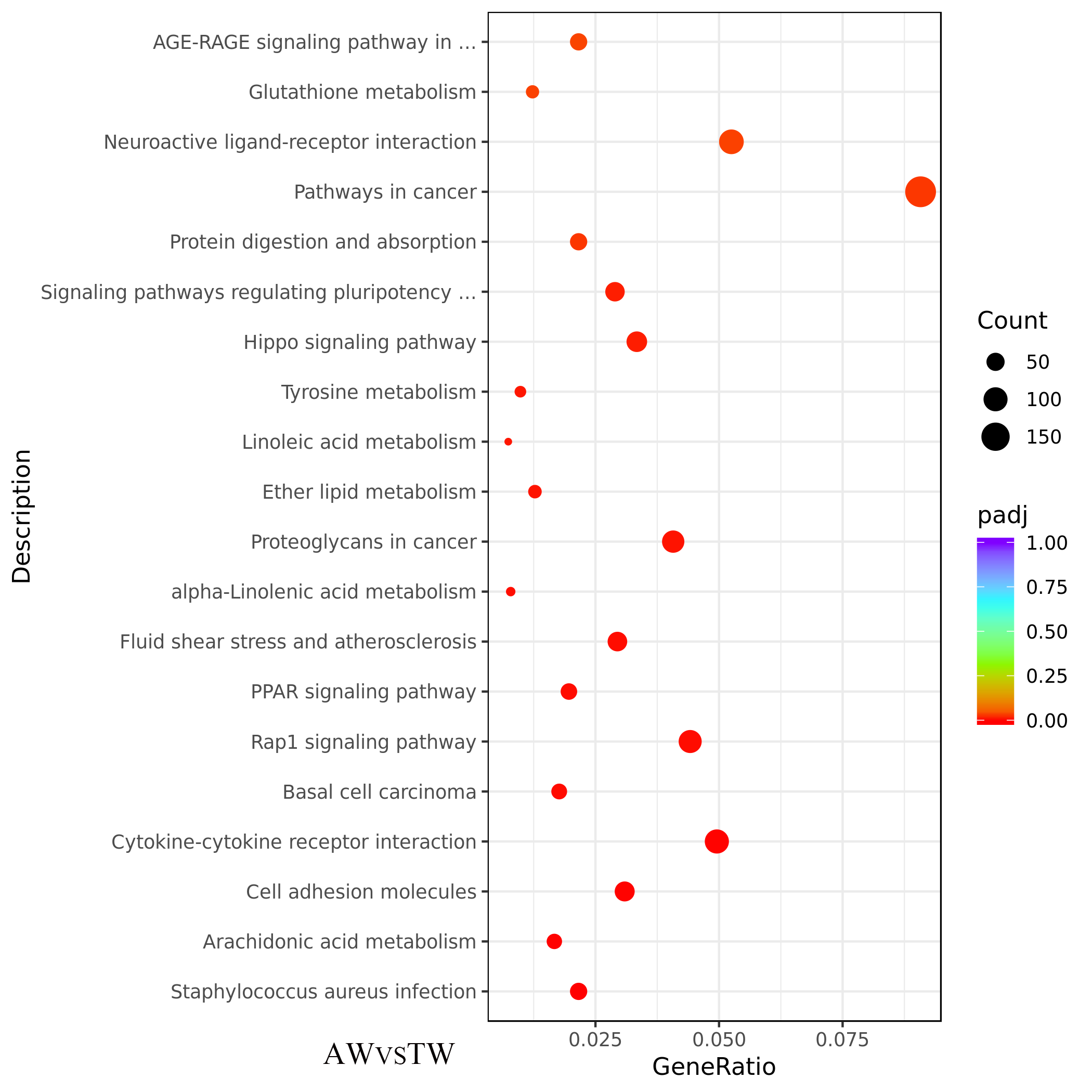

Supplement: Supplementary file 1 [file animals-12-03088-s001.zip › Supplementary/FigS6.png]

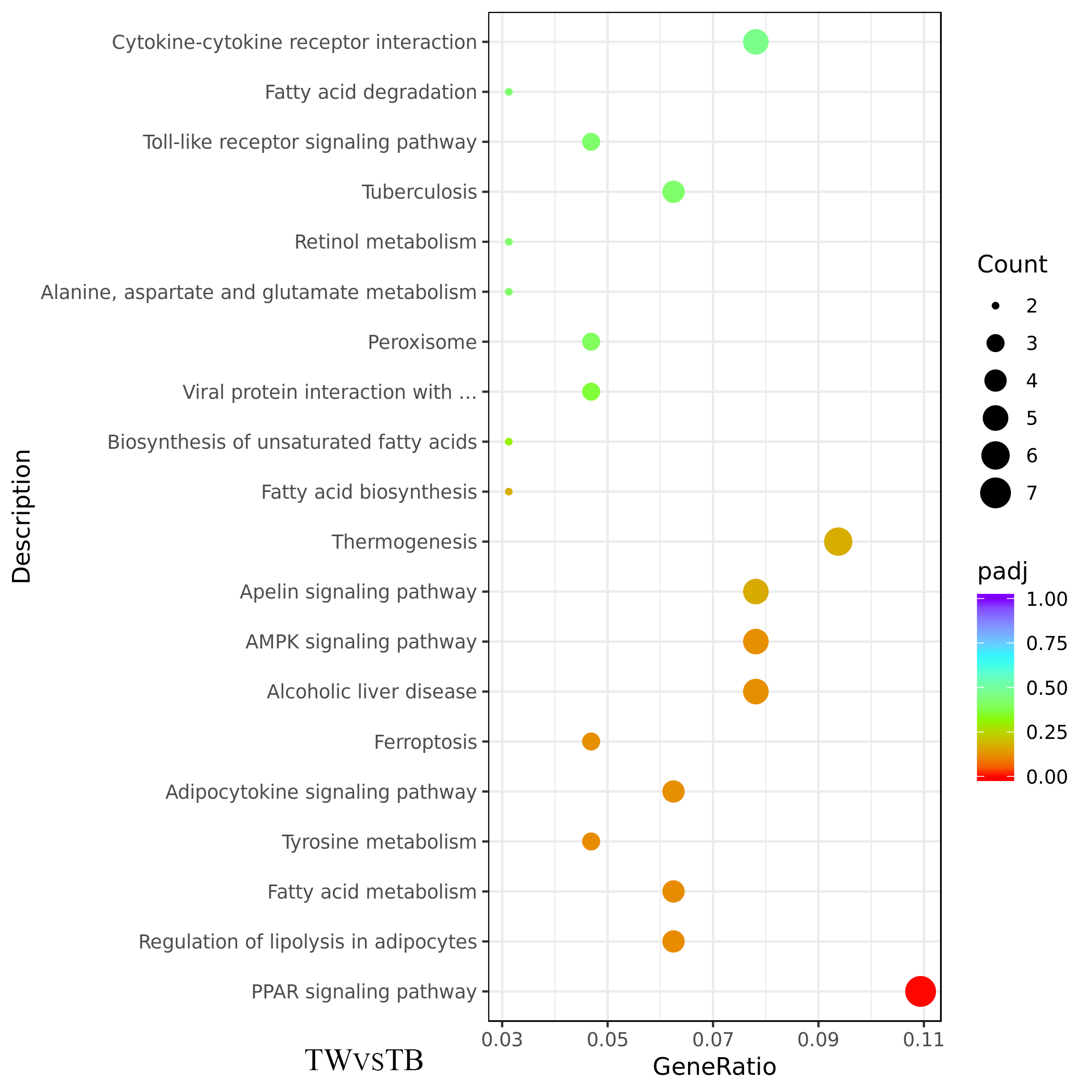

Supplement: Supplementary file 1 [file animals-12-03088-s001.zip › Supplementary/FigS7.png]

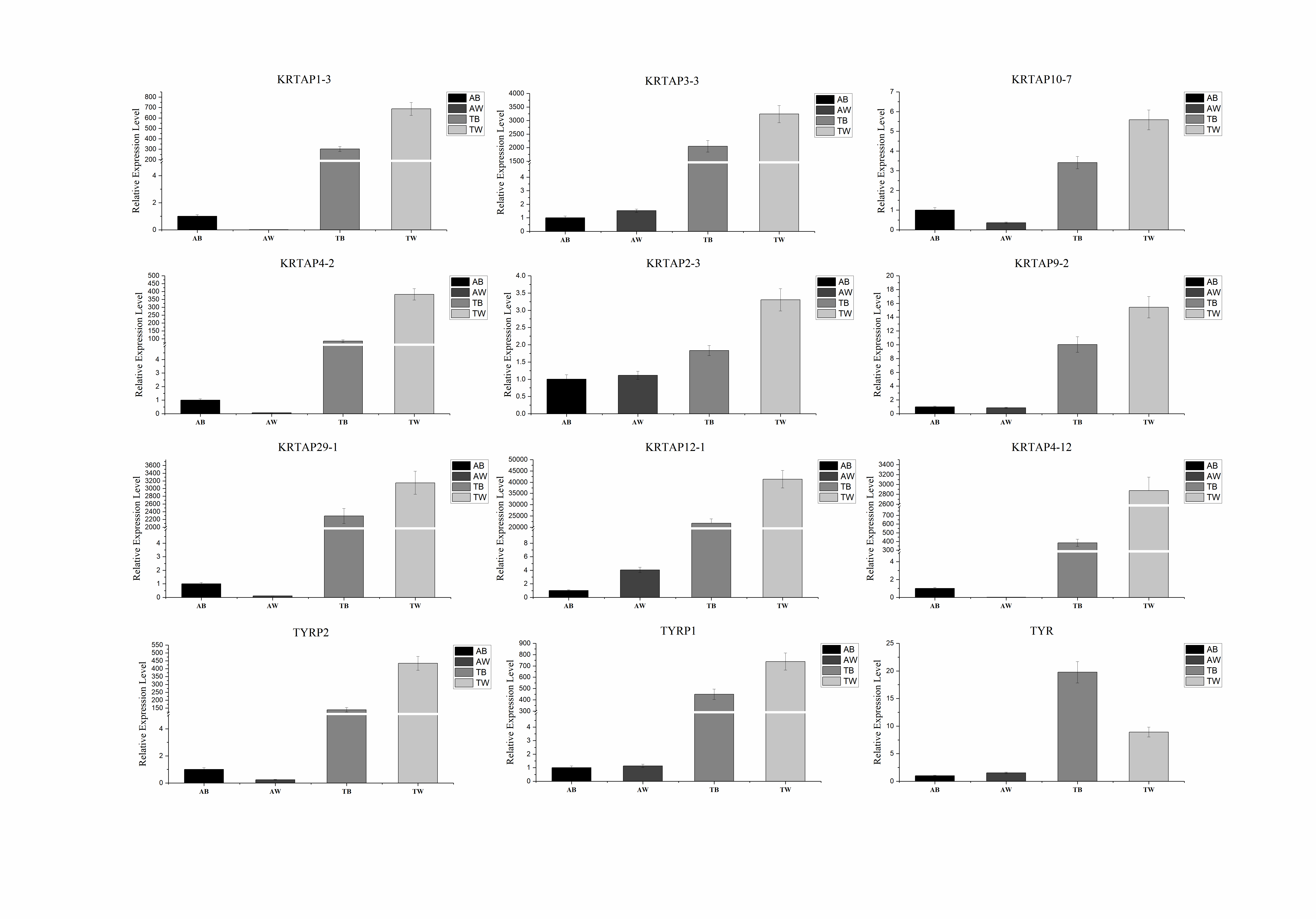

Supplement: Supplementary file 1 [file animals-12-03088-s001.zip › Supplementary/FigS8.jpg]
